# Supplementary figures and images for: Stability of SARS-CoV-2-Encoded Proteins and Their Antibody Levels Correlate with Interleukin 6 in COVID-19 Patients
Source: mSystems. 2022 May 18;7(3):e00058-22. doi: 10.1128/msystems.00058-22 (PMC9238396; doi:10.1128/msystems.00058-22)

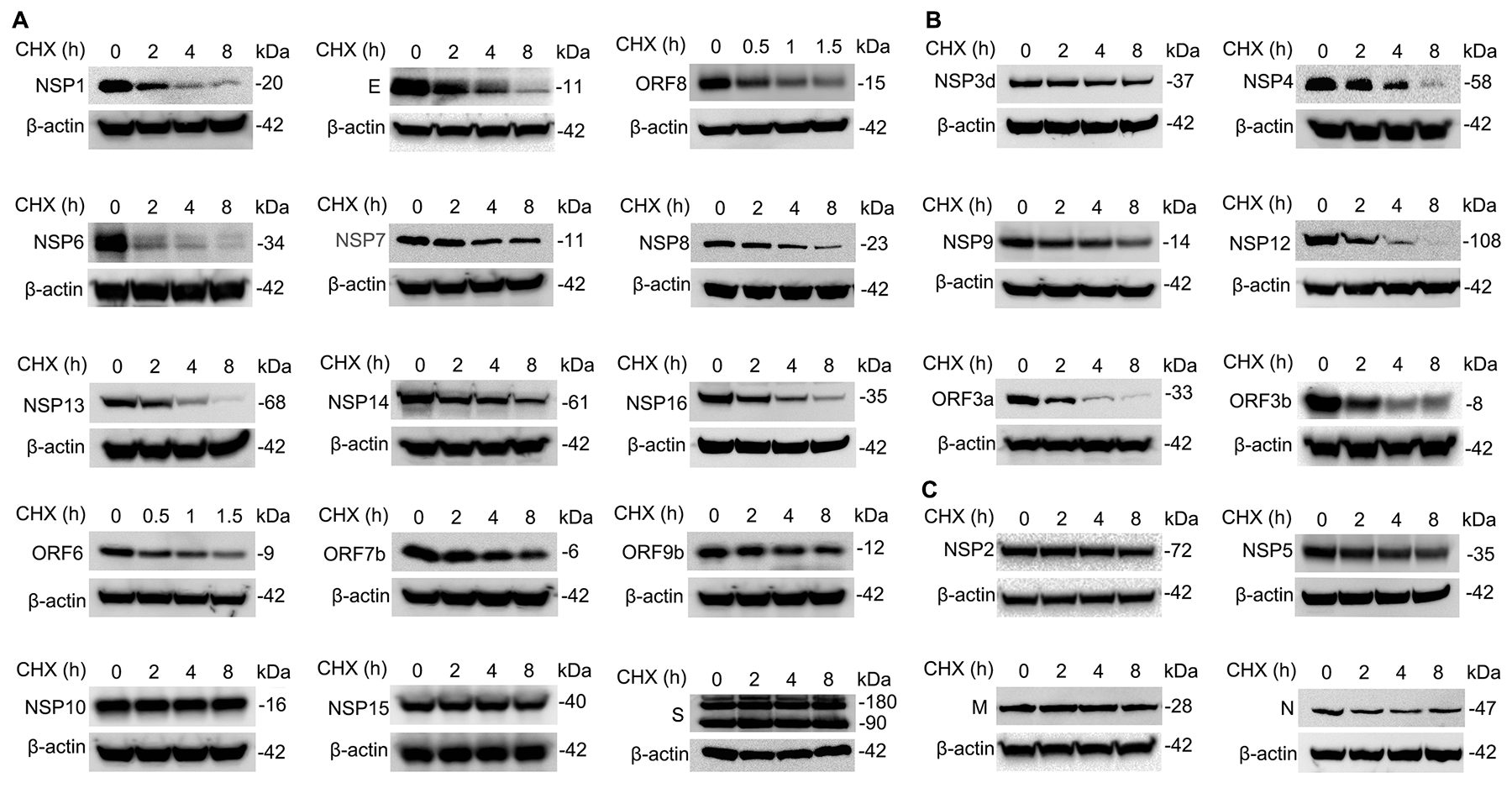

Supplement: FIG S1 [file msystems.00058-22-s0004.tif]

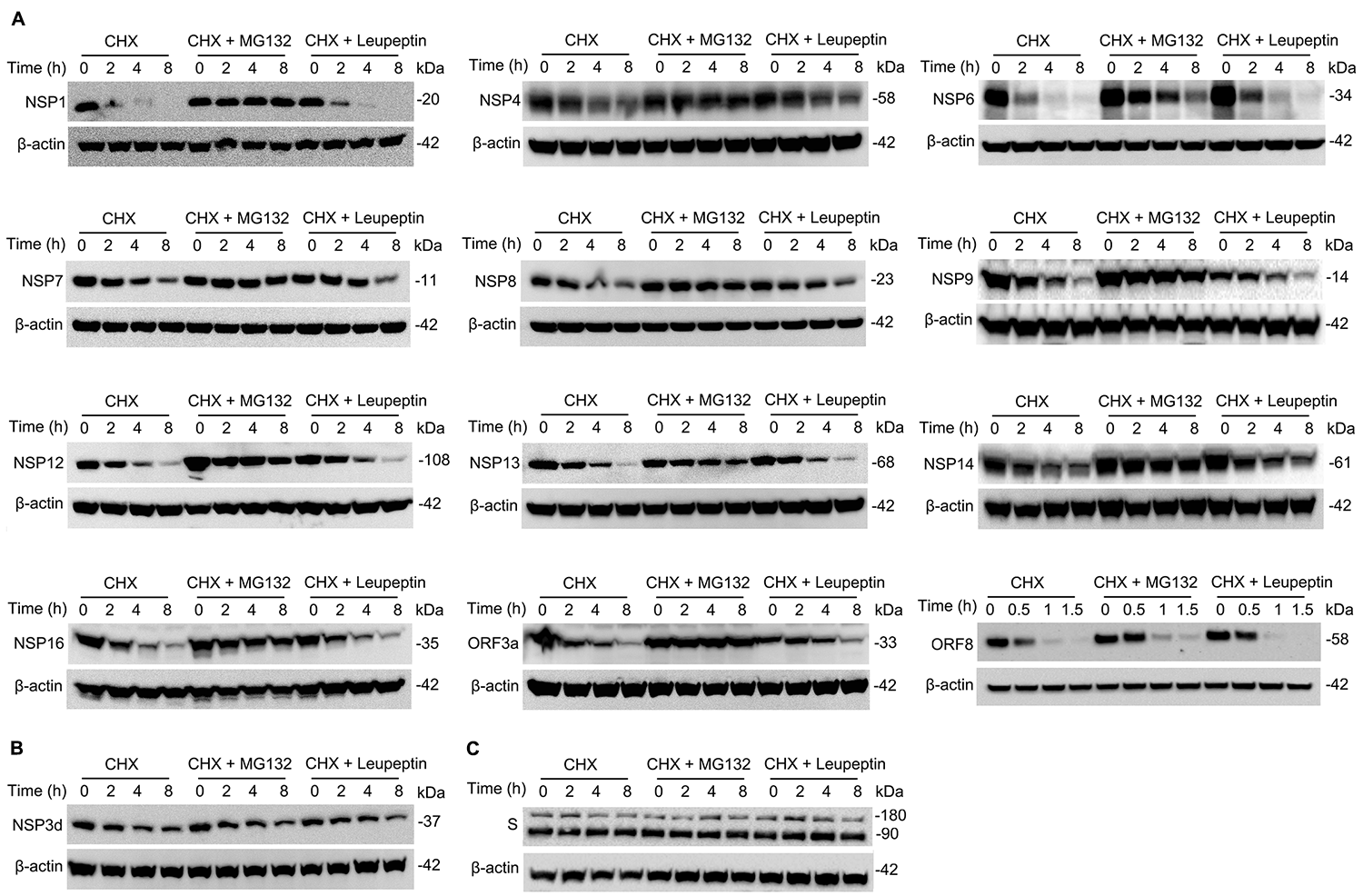

Supplement: FIG S2 [file msystems.00058-22-s0005.tif]
