# Supplementary material for: Stability of SARS-CoV-2-Encoded Proteins and Their Antibody Levels Correlate with Interleukin 6 in COVID-19 Patients
Source: mSystems. 2022 May 18;7(3):e00058-22. doi: 10.1128/msystems.00058-22 (PMC9238396; doi:10.1128/msystems.00058-22)
Supplement: TABLE S3 [file msystems.00058-22-s0003.docx]

**TABLE S3. Half-life of SARS-CoV-2 proteins**.

| No. | Plasmid name | Amino acids  (No.) | Molecular weight  (kDa) | half-life  (h) |
| --- | --- | --- | --- | --- |
| 1 | pETM33_Nsp1 | 180 | 20 | 1.57 |
| 2 | pDONR223 SARS-CoV-2 NSP2 | 638 | 72 | ＞ 8.00 |
| 3 | pETM33_Nsp3d_Ub2_PLPro | 311 | 37 | 7.72 |
| 4 | pDONR223 SARS-CoV-2 NSP4 | 500 | 58 | 4.51 |
| 5 | pDONR223 SARS-CoV-2 NSP5 | 306 | 35 | ＞ 8.00 |
| 6 | pDONR223 SARS-CoV-2 NSP6 | 290 | 34 | 1.81 |
| 7 | pDONR223 SARS-CoV-2 NSP7 | 83 | 11 | 6.05 |
| 8 | pDONR223 SARS-CoV-2 NSP8 | 198 | 23 | 7.04 |
| 9 | pDONR223 SARS-CoV-2 NSP9 | 113 | 14 | 7.87 |
| 10 | pDONR223 SARS-CoV-2 NSP10 | 139 | 16 | ＞ 8.00 |
| 11 | pLVX-EF1alpha-SARS-CoV-2-nsp11-2xStrep-IRES-Puro | 13 | NA | NA |
| 12 | pDONR223 SARS-CoV-2 NSP12 | 932 | 108 | 3.16 |
| 13 | pDONR223 SARS-CoV-2 NSP13 | 601 | 68 | 3.26 |
| 14 | pDONR223 SARS-CoV-2 NSP14 | 527 | 61 | 6.61 |
| 15 | pDONR223 SARS-CoV-2 NSP15 | 346 | 40 | ＞ 8.00 |
| 16 | pDONR223 SARS-CoV-2 NSP16 | 298 | 35 | 3.22 |
| 17 | pDONR223 SARS-CoV-2 ORF3b | 58 | 8 | 1.74 |
| 18 | pDONR223 SARS-CoV-2 ORF6 | 61 | 9 | 0.94 |
| 19 | pDONR223 SARS-CoV-2 ORF7b | 43 | 6 | 6.57 |
| 20 | pDONR223 SARS-CoV-2 ORF8 | 121 | 15 | 0.48 |
| 21 | pDONR223 SARS-CoV-2 ORF9b | 97 | 12 | 7.68 |
| 22 | pDONR207 SARS-CoV-2 ORF3a | 275 | 33 | 2.67 |
| 23 | pDONR207 SARS-CoV-2 ORF7a | 121 | NA | NA |
| 24 | pcDNA3.1-SARS-CoV-2-Spike | 1273 | 180 | ＞ 8.00 |
| 25 | pLVX-EF1alpha-SARS-CoV-2-E-2xStrep-IRES-Puro | 75 | 11 | 3.92 |
| 26 | pLVX-EF1alpha-SARS-CoV-2-M-2xStrep-IRES-Puro | 222 | 28 | ＞ 8.00 |
| 27 | pLVX-EF1alpha-SARS-CoV-2-N-2xStrep-IRES-Puro | 419 | 47 | ＞ 8.00 |
| 28 | pLXV-EF1alpha-2xStrep-SARS-CoV-2-orf9c-IRES-Puro | 73 | NA | NA |
| 29 | pLVX-EF1alpha-SARS-CoV-2-orf10-2xStrep-IRES-Puro | 38 | NA | NA |

*: NA denotes not assayed.
