# Supplementary material for: Stability of SARS-CoV-2-Encoded Proteins and Their Antibody Levels Correlate with Interleukin 6 in COVID-19 Patients
Source: mSystems. 2022 May 18;7(3):e00058-22. doi: 10.1128/msystems.00058-22 (PMC9238396; doi:10.1128/msystems.00058-22)
Supplement: TABLE S1 [file msystems.00058-22-s0001.docx]

**TABLE S1. Information of SARS-CoV-2 plasmids**.

| **Plasmid** | **From** | **Cat.#** | **Website** | **RRID** |
| --- | --- | --- | --- | --- |
| pETM33_Nsp1 | Ylva Ivarsson | 156465 | http://n2t.net/addgene:156465 | Addgene_156465 |
| pDONR207 SARS-CoV-2 NSP3 | Ylva Ivarsson | 141257 | http://n2t.net/addgene:141257 | Addgene_141257 |
| pDONR207 SARS-CoV-2 NSP3 | Fritz Roth | 141257 | http://n2t.net/addgene:141257 | Addgene_141257 |
| pDONR207 SARS-CoV-2 ORF3A | Fritz Roth | 141271 | http://n2t.net/addgene:141271 | Addgene_141271 |
| pDONR207 SARS-CoV-2 ORF7A | Fritz Roth | 141276 | http://n2t.net/addgene:141276 | Addgene_141276 |
| pDONR223 SARS-CoV-2 NSP2 | Fritz Roth | 141256 | http://n2t.net/addgene:141256 | Addgene_141256 |
| pDONR223 SARS-CoV-2 NSP4 | Fritz Roth | 141258 | http://n2t.net/addgene:141258 | Addgene_141258 |
| pDONR223 SARS-CoV-2 NSP5 | Fritz Roth | 141259 | http://n2t.net/addgene:141259 | Addgene_141259 |
| pDONR223 SARS-CoV-2 NSP6 | Fritz Roth | 141260 | http://n2t.net/addgene:141260 | Addgene_141260 |
| pDONR223 SARS-CoV-2 NSP7 | Fritz Roth | 141261 | http://n2t.net/addgene:141261 | Addgene_141261 |
| pDONR223 SARS-CoV-2 NSP8 | Fritz Roth | 141262 | http://n2t.net/addgene:141262 | Addgene_141262 |
| pDONR223 SARS-CoV-2 NSP9 | Fritz Roth | 141263 | http://n2t.net/addgene:141263 | Addgene_141263 |
| pDONR223 SARS-CoV-2 NSP10 | Fritz Roth | 141264 | http://n2t.net/addgene:141264 | Addgene_141264 |
| pDONR223 SARS-CoV-2 NSP12 | Fritz Roth | 141265 | http://n2t.net/addgene:141265 | Addgene_141265 |
| pDONR223 SARS-CoV-2 NSP13 | Fritz Roth | 141266 | http://n2t.net/addgene: 141266 | Addgene_141266 |
| pDONR223 SARS-CoV-2 NSP14 | Fritz Roth | 141267 | http://n2t.net/addgene: 141267 | Addgene_141267 |
| pDONR223 SARS-CoV-2 NSP15 | Fritz Roth | 141268 | http://n2t.net/addgene: 141268 | Addgene_141268 |
| pDONR223 SARS-CoV-2 NSP16 | Fritz Roth | 141269 | http://n2t.net/addgene: 141269 | Addgene_141269 |
| pDONR223 SARS-CoV-2 ORF3B | Fritz Roth | 141272 | http://n2t.net/addgene: 141272 | Addgene_141272 |
| pDONR223 SARS-CoV-2 ORF6 | Fritz Roth | 141275 | http://n2t.net/addgene: 141275 | Addgene_141275 |
| pDONR223 SARS-CoV-2 ORF7B | Fritz Roth | 141277 | http://n2t.net/addgene: 141277 | Addgene_141277 |
| pDONR223 SARS-CoV-2 ORF8 | Fritz Roth | 141278 | http://n2t.net/addgene: 141278 | Addgene_141278 |
| pDONR223 SARS-CoV-2 ORF9B | Fritz Roth | 141280 | http://n2t.net/addgene: 141280 | Addgene_141280 |
| pETM33_Nsp1 | Ylva Ivarsson | 156465 | http://n2t.net/addgene: 156465 | Addgene_156465 |
| pETM33_Nsp3d_Ub2_PLPro | Ylva Ivarsson | 156472 | http://n2t.net/addgene: 156472 | Addgene_156472 |
| pETM33_Nsp5_Mpro | Ylva Ivarsson | 156475 | http://n2t.net/addgene: 156475 | Addgene_156475 |
| pLVX-EF1alpha-SARS-CoV-2-E-2xStrep-IRES-Puro | Nevan Krogan | 141385 | http://n2t.net/addgene: 141385 | Addgene_141385 |
| pLVX-EF1alpha-SARS-CoV-2-M-2xStrep-IRES-Puro | Nevan Krogan | 141386 | http://n2t.net/addgene: 141386 | Addgene_ 141386 |
| pLVX-EF1alpha-SARS-CoV-2-N-2xStrep-IRES-Puro | Nevan Krogan | 141391 | http://n2t.net/addgene: 141391 | Addgene_ 141391 |
| pcDNA3.1-SARS2-Spike | Fang Li | 145032 | http://n2t.net/addgene: 145032 | Addgene_ 145032 |
