# Supplementary material for: Stability of SARS-CoV-2-Encoded Proteins and Their Antibody Levels Correlate with Interleukin 6 in COVID-19 Patients
Source: mSystems. 2022 May 18;7(3):e00058-22. doi: 10.1128/msystems.00058-22 (PMC9238396; doi:10.1128/msystems.00058-22)
Supplement: TABLE S2 [file msystems.00058-22-s0002.docx]

**TABLE S2. Primers for subcloning of SARS-CoV-2 genes.**

| **Genes** | **Forward (5’-3’)** | **Reverse (5’-3’)** |
| --- | --- | --- |
| NSP1 | cacc atg cac gtt cag ctg agc ctg | acc gtt ttt acg cag cag cac c |
| NSP2 | cacc atg tcc gcc gct aga ctg ac | ctg cag cac ggt gtg agg ggt g |
| NSP3d | cacc atg gag gtg cgc acc atc | ggt ggt ggt ata gct gtt ttc |
| NSP4 | cacc atg aag atc gtg aac aac | ctg cag cac ggc gct ggt g |
| NSP5 | cacc atg gag gtg cgc acc atc | ggt ggt ggt ata gct gtt ttc |
| Nsp6 | cacc atg agc gcc gtg aag aga acc | ctg cac ggt ggc cac ctt gat g |
| NSP7 | cacc atg agc aag atg tcc gac g | ctg cag ggt agc cct gtt gtc c |
| NSP8 | cacc atg gcc atc gct agc gag | ctg cag ctt cac ggc aga g |
| NSP9 | cacc atg aac aac gag ctg agc c | ctg cag cct cac ggt agc agc |
| NSP10 | cacc atg gct ggt aac gct act gag g | ctg cag cat agg ttc cct c |
| NSP12 | cacc atg tcc gcc gct aga ctg ac | ctg cag ctc ggt gtg agg ggt g |
| NSP13 | cacc atg gcc gtg ggc gct tgc tg | ctg cag ggt agc cac gtt cct gcg |
| NSP14 | cacc atg gct gag aac gtg acc g | ctg cag cct ggt gaa ggt gtt c |
| NSP15 | cacc atg tct ctg gag aac gtg gc | ctg cag ctt agg gta gaa ggt ttc |
| NSP16 | cacc atg tct tcc cag gct tgg c | ga ctg cac gac cac ttg ttg |
| Spike | cacc atg ttt gtc ttc ctg gtc ctg c | tgt ata gtg cag ttt gac gcc c |
| N | cacc atg agc gat aac ggc ccc | cgc ctg agt aga atc ggc tga gc |
| Orf3a | cacc atg gac ctg ttc atg aga atc | cag tgg cac gga ggt ggt ggt g |
| Orf3b | cacc atg gcc tac tgc tgg cgc tg | agg cca gca gca tct agc gaa g |
| Orf6 | cacc atg ttc cac ctg gtg gac | gtc gat ttc cat ggg ctg ttc c |
| Orf7b | cacc atg atc gag ctg agc ctg atc | agc gtg gca ggt ttc gtt gtg g |
| Orf8 | cacc atg aag ttc ctg gtg ttc ctg | gat gaa gtc cag cac cac acg c |
| Orf9b | cacc atg gac cca aag atc tct g | ctt cac ggt cac cac cac g |

*: The sequence of all the primers are in 5’-3’ order.
